# Supplementary material for: Multi-night cortico-basal recordings reveal mechanisms of NREM slow-wave suppression and spontaneous awakenings in Parkinson’s disease
Source: Nat Commun. 2024 Feb 27;15:1793. doi: 10.1038/s41467-024-46002-7 (PMC10899224; doi:10.1038/s41467-024-46002-7)
Supplement: Supplementary file 1 — Supplementary Information [file 41467_2024_46002_MOESM1_ESM.pdf]

Supplementary material
for
Multi-night cortico-basal recordings reveal mechanisms of NREM slow-wave suppression and spontaneous awakenings in Parkinson's disease

Anjum, et al., 2024

Supplementary Table 1: Sleep Statistics

	ON stimulation					OFF stimulation			
Participant ID	DYS	PD3	PD9	PD2	PD7	PD3	PD9	PD2	PD7
SO (min)	18.8±2.4	29.9±3.7	31.8±4.1	19.2±1.9	25.9±2.7	22.4	106.4	34.9	22.5
N1 (min)	23.5±2.7	33.5±2.7	28.1±2.5	35.7±3.0	38.3±1.8	17.5	33	47.9	25
N2 (min)	143.0±13.0	100.9±9.8	187.5±13.6	182.4±10.1	187.8±14.4	94.5	171.1	237.5	235.3
N3 (min)	99.5±6.1	205.9±10.3	36.2±5.3	54.2±5.8	71.7±3.5	151.4	22.5	75.8	61.2
REM (min)	55.8±6.3	112.8±8.0	121.4±17.4	59.5±5.4	91.5±12.0	140.6	72.5	62.8	92.8
N2+N3 (min)	242.8±13.0	306.8±14.4	223.7±14.1	236.6±9.3	259.5±14.0	245.9	193.5	313.3	296.5
WASO (min)	61.3±12.3	66.5±8.3	15.6±3.5	61.9±9.0	38.1±4.3	128.9	66.5	100.2	25.4
Wake events	23.6±2.8	32.5±2.0	12.9±2.0	18.0±2.2	36.9±3.2	19	12	31	17
TST (hours)	6.4±0.2	8.7±0.1	6.5±0.3	6.6±0.3	7.1±0.3	8.9	6.1	8.7	7.3
Total nights	9	11	10	12	11	1	1	1	1

SO = Time to Sleep onset; WASO= Wake after sleep onset; N1, N2, N3, REM, N2+N3 and WASO: total duration times per night in minutes; TST= total sleep time; Wake events = total wake after sleep events during one night. ON stimulation includes average sleep metrics for multiple nights of recordings at home. OFF stimulation includes a single night of at-home recording in the absence of stimulation.

Supplementary Table 2: N2/N3 NREM vs wakefulness classification (cortex)

	30 second epoch				5 second epoch			
Participant ID	PD3	PD9	PD2	PD7	PD3	PD9	PD2	PD7
Accuracy	92.4	96.1	95.7	90.2	89.4	94.2	92.1	86.7
AUC	96.6	98.8	98.5	96.1	94.2	97.8	96.7	93
Sensitivity	92.9	97.0	96	89.6	92.6	96.2	95	90.7
Specificity	91.9	95.1	95.4	90.8	86.1	92.3	89.2	82.7
PPV	92.0	95.2	95.5	90.7	87	92.6	89.8	83.9
NPV	92.9	96.9	96	89.7	92.1	96.1	94.7	89.9
Odds ratio	149.8	631.8	500.7	85.6	77.9	303.5	155.9	46.4
U-test p-value	0	0	0	0	0	0	0	0

Individual machine-learning model performance for N2/N3 NREM vs wakefulness binary classification using cortical data. PPV = positive predictive value, NPV = negative predictive value, U-test = Wilcoxon rank sum test, AUC = Area under the receiver operating characteristic curve.

Supplementary Table 3: N2/N3 NREM vs wakefulness classification (sub-cortex)

	30 second epoch				5 second epoch			
Participant ID	PD3	PD9	PD2	PD7	PD3	PD9	PD2	PD7
Accuracy	87.7	80.9	95.5	85.6	80.8	70.4	90.4	80.9
AUC	94.8	89.9	98.6	92.8	89.4	79.6	96	88.2
Sensitivity	86.3	79.9	95.4	84.4	87.5	90.5	91.4	85.5
Specificity	89.1	81.9	95.6	86.8	74.1	50.3	89.4	76.3
PPV	88.8	81.5	95.6	86.4	77.1	64.5	89.6	78.3
NPV	86.7	80.3	95.4	84.8	85.6	84.1	91.2	84
Odds ratio	51.8	18	448.6	35.6	20	9.6	90.2	18.9
U-test p-value	0	0	0	0	0	0	0	0

Individual machine-learning model performance for N2/N3 NREM vs wakefulness binary classification using subcortical data. PPV = positive predictive value, NPV = negative predictive value, U-test = Wilcoxon rank sum test, AUC = Area under the receiver operating characteristic curve.

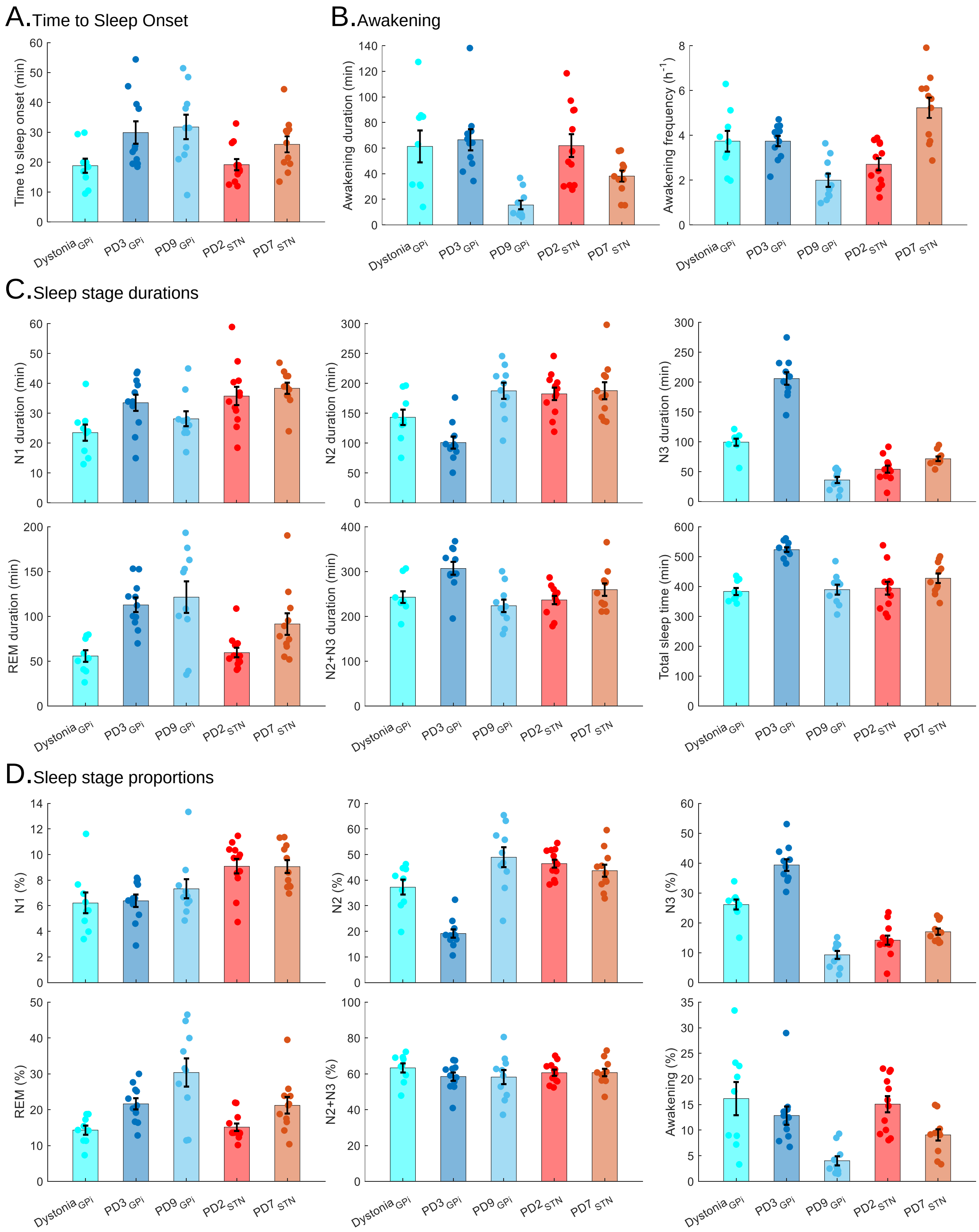

Supplemental Figure 1: Sleep statistics in ON stimulation. Sleep statistics (mean±SEM) for all 5 participants during overnight recordings with ON stimulation. **(A)** Time to sleep onset **(B)** Wake after sleep onset (awakening during the sleep) in total duration and frequency over one night **(C)** Total durations of all sleep stages (N1, N2, N3, N2/N3 NREM and REM) and total sleep time in minutes **(D)** Total proportion of sleep stages (N1, N2, N3, N2/N3 NREM, REM and Awakening). For all panels, n=12 (PD2); n=11(PD3); n=11 (PD7); n=10 (PD9); n=9 (Dystonia). Source data are provided as a Source Data file.

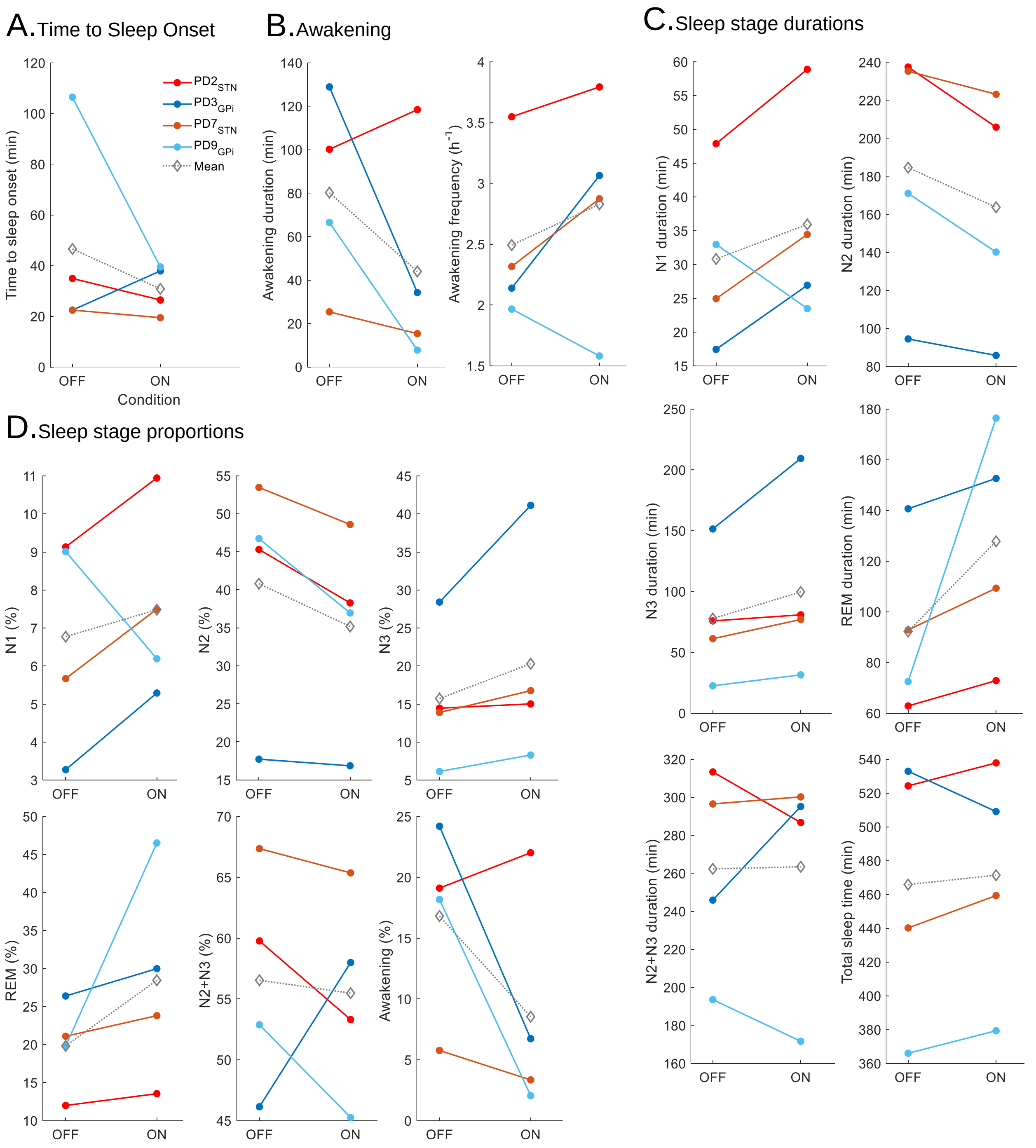

Supplemental Figure 2: ON vs OFF stimulation sleep statistics. Sleep statistics for all PD participants (n=4) during overnight recordings during consecutive one night ON and one night OFF stimulation conditions. **(A)** Time to sleep onset **(B)** Wake after sleep onset (awakening during the sleep) in total duration and frequency over one night **(C)** Total durations of all sleep stages (N1, N2, N3, N2/N3 NREM and REM) and total sleep time in minutes **(D)** Total proportion of sleep stages (N1, N2, N3, N2/N3 NREM, REM and Awakening). x-axes are stimulation conditions (ON/OFF) and the dashed gray lines show average across all PD participants. Source data are provided as a Source Data file.

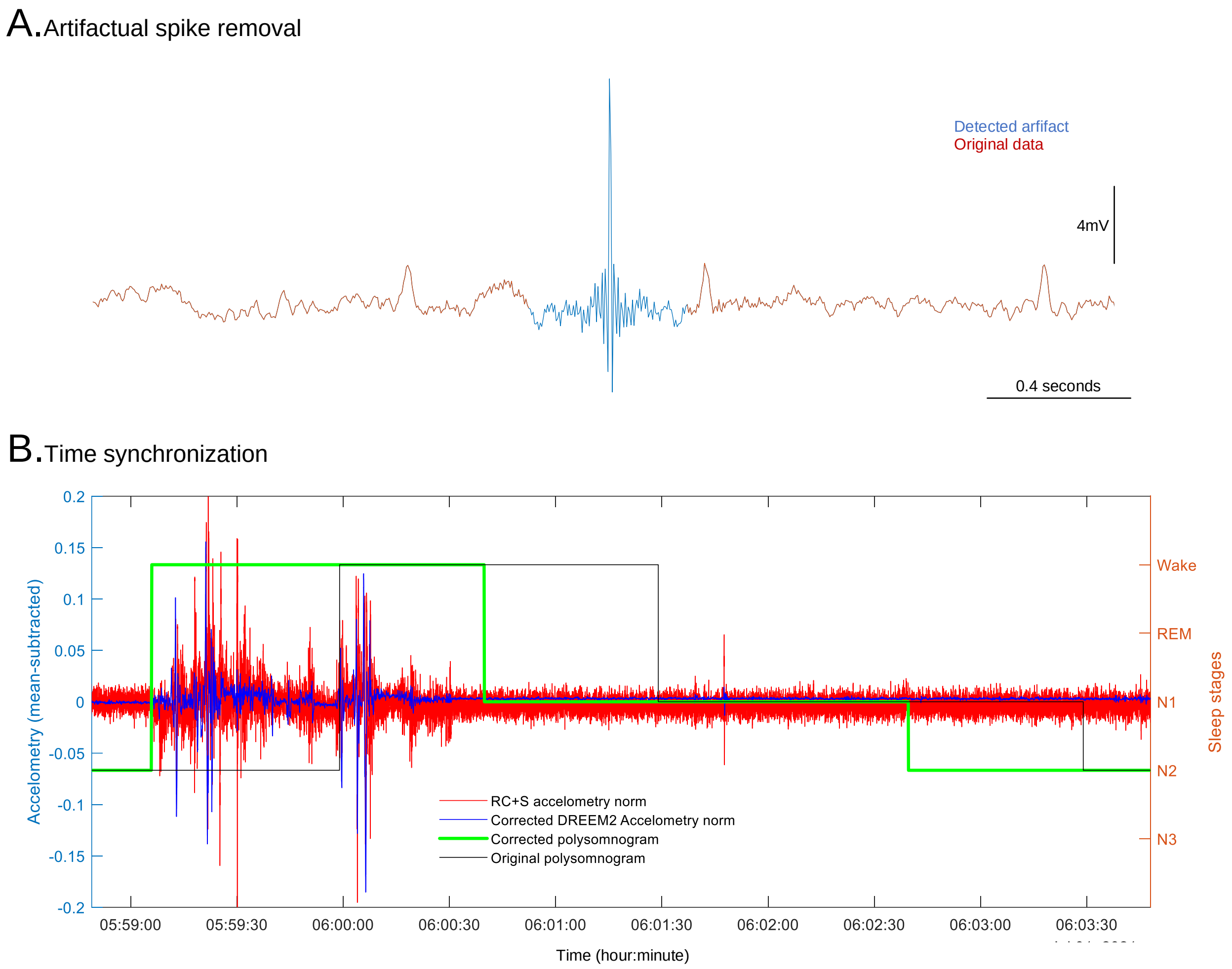

Supplemental Figure 3: Data processing procedures. (A) Example of the removal of the movement-related spike artifact from the RC+S field potential data. **(B)** Example of the time synchronization between polysomnogram from Dreem2 and intracranial data streams from RC+S devices using accelerometry data as references. Source data are provided as a Source Data file.

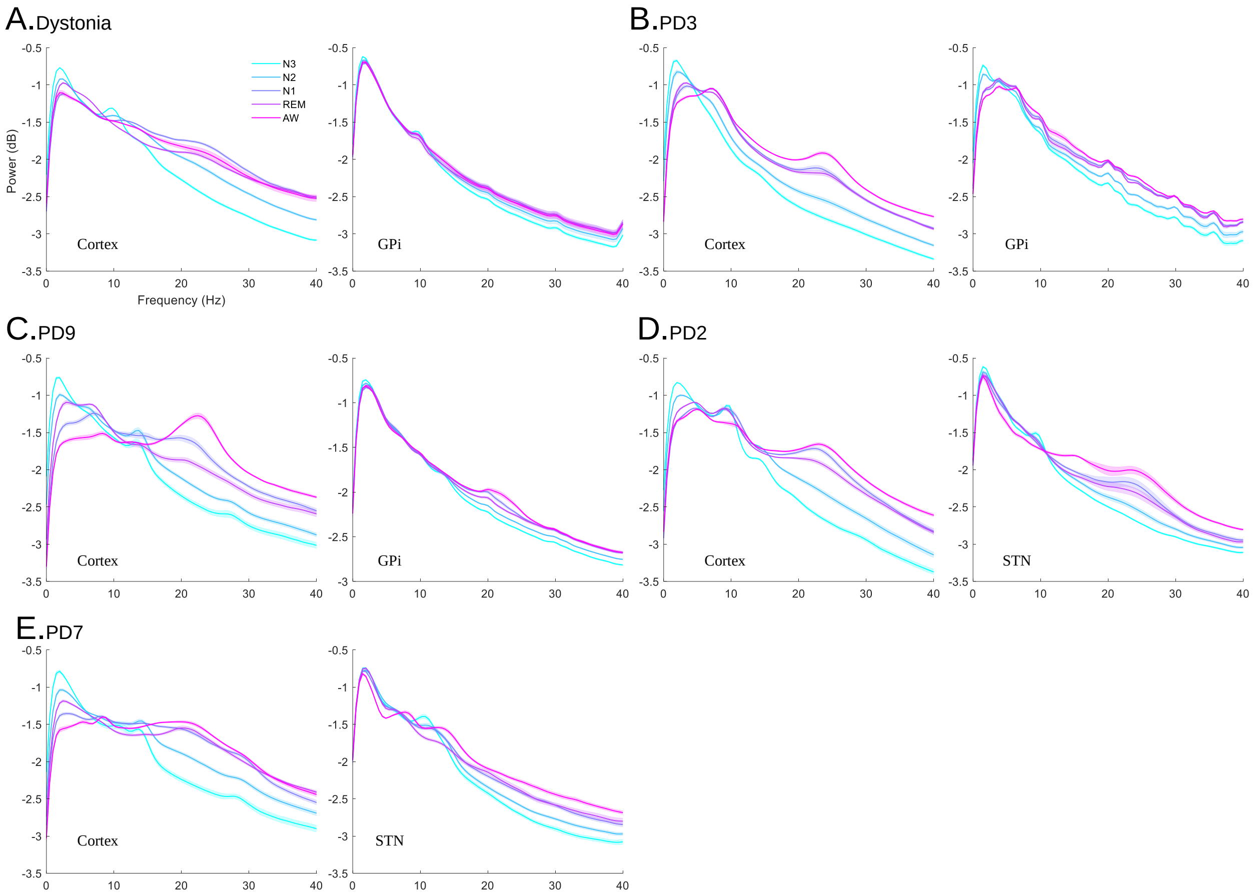

Supplemental Figure 4: Power spectrum of sleep stages. Spectral power (mean ± SEM; 5s epochs; averaged across each night; data pooled from both hemispheres) in cortex and subcortex for all sleep stages in each participant (n=5; ON stimulation). For all panels, n=12 (PD2); n=11(PD3); n=11 (PD7); n=10 (PD9); n=9 (Dystonia). Source data are provided as a Source Data file.

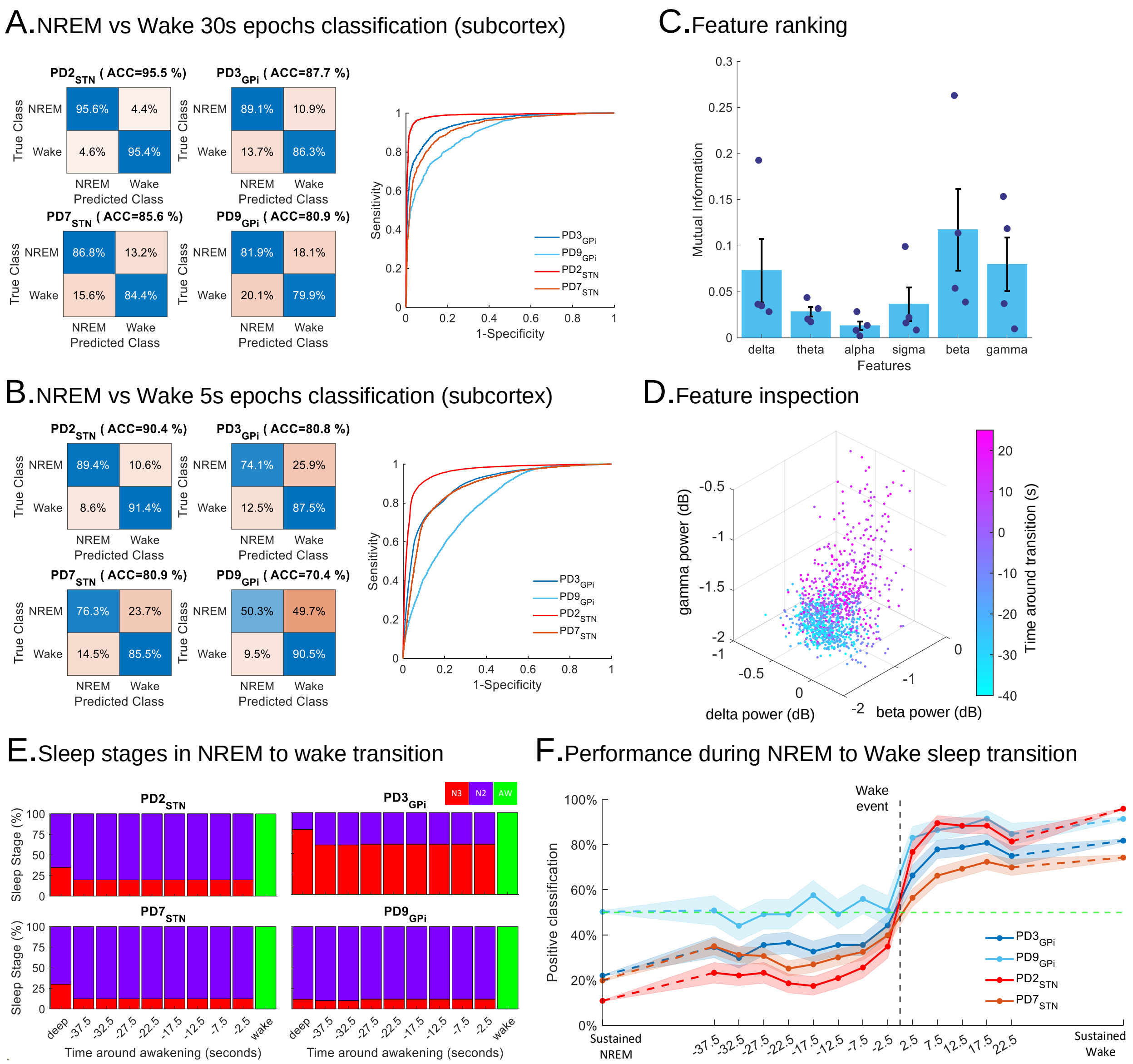

Supplemental Figure 5: Classification of N2/N3 NREM vs wakefulness with subcortical FP (A) Performance of patient-specific machine learning (ML) models for N2/N3 NREM vs wakefulness classification using participant-specific ML models for all PD patients (n=4) with classical 30s epoch window in terms of confusion matrices (*left*) and receiver operating characteristic (ROC) performance (*right*). **(B)** Same as A, for 5s epoch window. **(C)** Bandpower feature importance and ranking where x-axis shows 6 bandpower features and y-axis shows average mutual information between bandpower and N2/N3 NREM vs wake state across all PD participants (mean±SEM; n=4; each dot is one participant). 5s data epochs were utilized. **(D)** Depiction of the top three bandpower features (delta, beta, and gamma) in a scatter plot for N2/N3 NREM to wake after sleep transition. Color bar (*left*) shows the time around awakening in seconds. Data points represent 5-second epochs from a single PD participant (PD2). **(E)** Ratio of N2 and N3 sleep stages across all N2/N3 NREM to wake after sleep transitions for each PD participant. Data from all N2/N3 NREM to wake transition events were pooled for each participant. x-axis is the time around the wake event and y-axis shows the stack plot of N2 and N3 durations. **(F)** Performance of the ML models

trained on 5s epochs shown in B during N2/N3 NREM to wake after sleep transition. The x- axis represents the time around awakening and y-axis is wake classification across all transitions of the participant (mean±SEM) with n=86 (PD2), n=104 (PD3), n=163 (PD7), and n=59 (PD9). The vertical black dashed line shows awakening time and the horizontal green dashed line represents 50% average wake detection. For all panels, left and right side data were pooled. Source data are provided as a Source Data file.

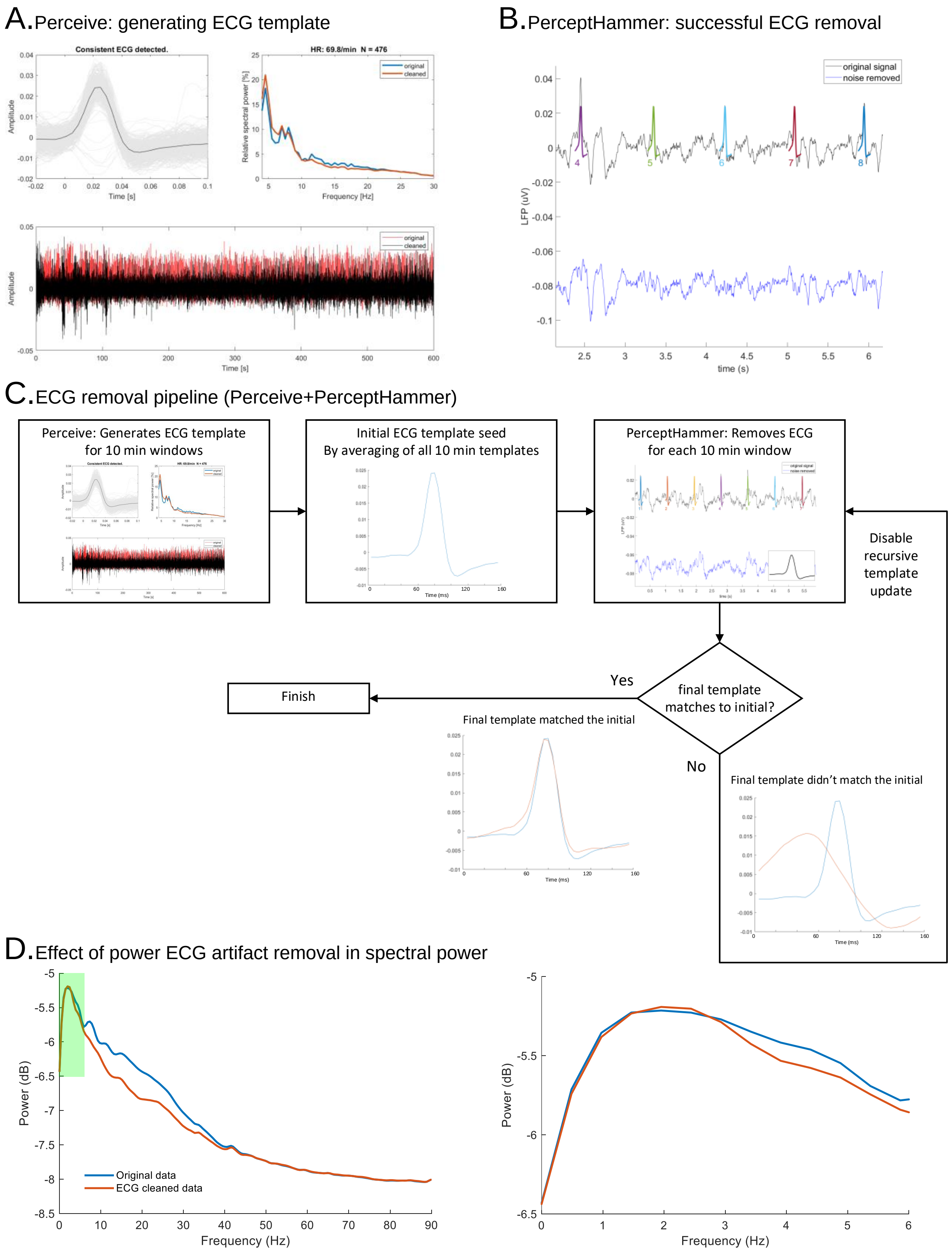

Supplemental Figure 6: ECG artifact removal procedure. (A) Illustration of ECG template generation by Perceive method. The top left panel shows the ECG template found in the data, the top right panel shows power spectrum comparison between pre and post ECG removal done by Perceive and the bottom panel

shows pre and post ECG removal time series comparison. **(B**) Illustration of ECG artifact removal by PerceptHammer method with initial template seed provided by Perceive. **(C)** Complete ECG artifact removal pipeline implemented in this study. Perceive method generates the initial ECG template seed. PerceptHammer attempts to remove ECG artifacts with default mode. If the final updated template doesn't sufficiently match with the initial template (maximum normalized cross-correlation < 0.9) then re-run PerceptHammer with recursive template update disabled. **(D)** Spectral power density comparison of the original subcortical data over one night from one PD participant (blue; Pre-ECG removal) and cleaned data (red: Post-ECG removal) over full frequency range (*left*; 0-90 Hz) and over low-frequency range (*right*; 0-6 Hz) showing minimal loss of the low-frequency spectral contents of ECG removal procedure. Major spectral changes following artifact removal were in a broad range (10-40 Hz). Of note, ECG artifacts have a broad spectral content due to their sharpness in time domain. These changes are consistent with the pre and post-ECG removal spectral changes observed by Hammer et al. (2022). Source data are provided as a Source Data file.

Study Protocol Documentation

This sleep research is a sub-study of a wider research project including both daytime neurophysiology and closed-loop deep brain stimulation. Therefore, below we describe both the parent study protocol information and also the sleep sub-study specific information (where different).

Type of trial: Parent trial - Interventional
Sleep sub-study – Observational

Registration site and number: *https://clinicaltrials.gov/study/NCT03582891*, *NCT03582891*

First posted date: 2018-07-11

Study start date: 2018-10-01

Completion date: 2028-03

Number of patients intended to be recruited: 25 (20 with Parkinson's disease and 5 with cervical dystonia)

Number of patients in sleep sub-study: 5 (4 patient with Parkinson's disease and 1 patient with cervical dystonia)

Exclusion and inclusion criteria:

Inclusion Criteria

Parkinson's Disease:

1. Ability to give informed consent for the study
2. Movement disorder symptoms that are sufficiently severe, in spite of best medical therapy, to warrant surgical implantation of deep brain stimulators according to standard clinical criteria
3. Patient has requested surgical intervention with deep brain stimulation for their disorder
4. No MR abnormalities that suggest an alternative diagnosis or contraindicate surgery
5. Absence of significant cognitive impairment (score of 20 or greater on the Montreal Cognitive Assessment (MoCA),
6. Signed informed consent
7. Ability to comply with study follow-up visits for brain recording, testing of adaptive stimulation, and clinical assessment.
8. Age 21-75 (for STN patients, minimum age is 25)
9. Diagnosis of idiopathic PD with duration of motor symptoms for 4 years or greater
10. Patient has undergone appropriate therapy with oral medications with inadequate relief as determined by a movement disorders neurologist, and has had stable doses of antiparkinsonian medications for 30 days prior to baseline assessment.

11. UPDRS-III score off medication between 20 and 80 and an improvement of at least 30% in the baseline UPDRS-III on medication score, compared to the baseline off-medication score, and motor fluctuations with at least 2 hours per day of on time without dyskinesia or with non-bothersome dyskinesia.

OR Patients with tremor-dominant PD (a tremor score of at least 2 on a UPDRS-III sub-score for tremor), treatment resistant, with significant functional disability despite maximal medical management

Dystonia:

1. Ability to give informed consent for the study
2. Movement disorder symptoms that are sufficiently severe, in spite of best medical therapy, to warrant surgical implantation of deep brain stimulators according to standard clinical criteria
3. Patient has requested surgical intervention with deep brain stimulation for their disorder
4. No MR abnormalities that suggest an alternative diagnosis or contraindicate surgery
5. Absence of significant cognitive impairment (score of 20 or greater on the Montreal Cognitive Assessment (MoCA)
6. Signed informed consent
7. Ability to comply with study follow-up visits for brain recording, testing of adaptive stimulation, and clinical assessment.
8. Age 21-75
9. Diagnosis of Isolated dystonia, which may be focal cervical, segmental craniocervical, or generalized forms.
10. Stable doses of anti-dystonia medications (such as trihexyphenydil, Baclofen, or clonazepam) for at least 30 days prior to baseline assessment
11. For dystonia patients with craniofacial and cervical involvement, prior treatment with botulinum toxin with failure to adequately control dystonia symptoms.

<u>Exclusion Criteria</u>

Parkinson's Disease:

1. Coagulopathy, anticoagulant medications, uncontrolled hypertension, history of seizures, heart disease, or other medical conditions considered to place the patient at elevated risk for surgical complications
2. Evidence of a psychogenic movement disorder: Motor symptoms that remit with suggestion or "while unobserved", symptoms that are inconsistent over time or incongruent with clinical condition, plus other manifestation such as "false" signs, multiple somatizations, or obvious psychiatric disturbance.
3. Pregnancy: all women of child bearing potential will have a negative urine pregnancy test prior to undergoing their surgical procedure.
4. Significant untreated depression (BDI-II score >20) History of suicidal attempt or active suicidal ideation (Yes to #2-5 on C-SSRS)
5. Any personality or mood symptoms that study personnel believe will interfere with study requirements.

6. Subjects who require ECT, rTMS or diathermy
7. Implanted stimulation systems such as; cochlear implant, pacemaker, defibrillator, neurostimulator or metallic implant
8. Previous cranial surgery
9. Drug or alcohol abuse
10. Meets criteria for Parkinson's disease with mild cognitive impairment (PD-MCI). These criteria are: performance of more than two standard deviations below appropriate norms, for tests from two or more of these five cognitive domains: attention, executive function, language, memory, and visuospatial tests.

Dystonia:

1. Coagulopathy, anticoagulant medications, uncontrolled hypertension, history of seizures, heart disease, or other medical conditions considered to place the patient at elevated risk for surgical complications
2. Evidence of a psychogenic movement disorder: Motor symptoms that remit with suggestion or "while unobserved", symptoms that are inconsistent over time or incongruent with clinical condition, plus other manifestation such as "false" signs, multiple somatizations, or obvious psychiatric disturbance.
3. Pregnancy: all women of child bearing potential will have a negative urine pregnancy test prior to undergoing their surgical procedure.
4. Significant untreated depression (BDI-II score >20) History of suicidal attempt or active suicidal ideation (Yes to #2-5 on C-SSRS)
5. Any personality or mood symptoms that study personnel believe will interfere with study requirements.
6. Subjects who require ECT, rTMS or diathermy
7. Implanted stimulation systems such as; cochlear implant, pacemaker, defibrillator, neurostimulator or metallic implant
8. Previous cranial surgery
9. Drug or alcohol abuse

Primary endpoints and timeframe (Parent trial):

- Duration of 'on' stimulation time without dyskinesia from motor diaries in adaptive compared to standard open loop stimulation. (Parkinson's disease patients) [Time Frame: Comparison will use data from the testing of open and closed-loop stimulation during chronic adaptive DBS testing at home.]
    - Duration of 'on' stimulation time without dyskinesia in adaptive compared to standard open loop stimulation determined from the patients' motor diaries. The self-report motor diary is a validated method to capture this information. Every half-hour, patients indicate in this diary which of 4 categories (on, on with troubling dyskinesia, off, or asleep) best reflected their predominant symptoms for the prior 30 minutes. Patients will complete this diary for 3 consecutive days. The total time spent in the 'on' state without troubling

dyskinesia will then be summed and averaged over 3 days for all three conditions (baseline, open-loop stimulation and closed-loop stimulation).

- The Burke-Fahn-Marsden Dystonia Rating Scale-Movement aDBS testing compared to pre-operative baseline(Dystonia Patients) [Time Frame: Comparison will use data from the testing of open and closed-loop stimulation during chronic adaptive DBS testing at home.]
    - This scales evaluates dystonia in nine body areas, including eyes, mouth, speech and swallowing, neck, trunk, and right and left arm and leg. The maximal total score is 120 - a higher score means worsening symptoms. Investigators will compare the dystonia symptoms and functional disability during adaptive stimulation compared to preoperative baseline.
- Toronto Western Spasmodic Torticollis Rating Scale during aDBS testing compared to pre-operative baseline (Dystonia Patients) [Time Frame: Comparison will use data from the testing of open and closed-loop stimulation during chronic adaptive DBS testing at home.]
    - This is a standardized scale to measure the severity, disability, and pain associated with cervical dystonia. The motor severity subscale consists of 10 items, with variable scaling and weighting. It also includes a disability scale with six items,and a pain scale with three items. The total score is the sum of each of the subscales. A higher score indicates greater disability.
- Karolinska Sleepiness Scale [Time Frame: Through study completion, up to 4 years]
    - This is a standardized scale for measuring sleepiness
- Psychomotor vigilance task (PVT) [Time Frame: Through study completion, up to 4 years]
    - This is a standardized behavioral task for measure alertness and attention
- Positive and Negative Affect Schedule (PANAS-SF) [Time Frame: Through study completion, up to 4 years]
    - This is a standardized mood questionnaire

Submitted: 2021-08-11
Primary completion (estimated): 2025-07-01

Secondary outcomes and timeframe (Parent trial):

- The Unified Parkinsons Disease Rating Scale (UPDRS) III scores off of medication in adaptive compared to standard open-loop stimulation. (Parkinson's disease patients) [Time Frame: Comparison will use data from the testing of open and closed-loop stimulation during chronic adaptive DBS testing at home.]
    - The UPDRS III is a motor rating scale. Investigators will compare the severity motor symptoms while the patient is off of Parkinsoniae (UPDRS) III scores off of medication in adaptive compared to standard open-loop stimulation. (Parkinson's disease patients)n medications in conventional (open-loop) versus adaptive (closed-loop) DBS.
- Schwab England scale in adaptive compared to standard open loop stimulation. (Parkinson's disease patients) [Time Frame: Comparison will use data from the testing of open and closed-loop stimulation during chronic adaptive DBS testing at home.]

    - This scale estimates the abilities of individuals living with Parkinson's Disease relative to a completely independent situation. Investigators will use it to compare the abilities of daily living in subjects during the open-loop and adaptive stimulation trial.
- Hoehn and Yahr Staging in the medication 'on' state in adaptive compared to standard open loop stimulation. (Parkinson's disease patients) [Time Frame: Comparison will use data from the testing of open and closed-loop stimulation during chronic adaptive DBS testing at home.]
    - The Hoehn and Yahr scale are used to describe the progression of Parkinson's disease based upon the level of motor impairment. This scale only includes one score ranging from 1 to 5 where a higher score indicates a higher level of motor impairment. Investigators will compare the level of disease progression between the open-loop and adaptive stimulation conditions.
- The patient' quality of life report (PDQ-39) in adaptive compared to standard open loop stimulation. The PDQ39 yields a score between 0 to 100, where a higher score indicates more health problems. (Parkinson's disease patients) [Time Frame: Comparison will use data from the testing of open and closed-loop stimulation during chronic adaptive DBS testing at home.]
    - The PDQ 39 questionnaire has 39 questions to assess the patient's life quality including mobility, emotional state, and bodily comfort. Investigators will make a comparison of patients' life quality of life between open-loop and adaptive stimulation.
- Patient's Global Impression of Change (PGIC) in adaptive compared to standard open loop stimulation. (Parkinson's disease patients) [Time Frame: Comparison will use data from the testing of open and closed-loop stimulation during chronic adaptive DBS testing at home.]
    - Patient Global Impression of Changes measures the self-reported level of overall improvement (motor and non-motor symptoms) in a patient on a 1-7 scale. A one indicates no change and a 7 is the greatest level of improvement.
- Total Electric Energy Delivered (TEED) by the pulse generator in adaptive compared to standard open loop stimulation. (all patients) [Time Frame: Comparison will use data from the testing of open and closed-loop stimulation during chronic adaptive DBS testing at home.]
    - Investigators will compare the total charge delivered by the pulse generator between open-loop and adaptive stimulation to determine if there is a potential energy savings.
- Short form 36 Quality of Life measure (Dystonia Patients) [Time Frame: Comparison will use data from the testing of open and closed-loop stimulation during chronic adaptive DBS testing at home.]
    - The SF-36 is a measure of self-reported health status which is scored on a 0-100 scale. The lower the score the more disability a patient experiences.
- Patient Global Impression of Change (Dystonia Patients) [Time Frame: Comparison will use data from the testing of open and closed-loop stimulation during chronic adaptive DBS testing at home.]
    - Patient Global Impression of Changes measures the self-reported level of overall improvement (motor and non-motor symptoms) in a patient on a 1-7 scale. A one indicates no change and a 7 is the greatest level of improvement.
- Resting state EEG Recording [Time Frame: Through study completion, up to 4 years]
    - Resting state cortical power will be analyzed as a surrogate marker of alertness

Primary endpoints and timeframe (Sleep substudy):

1. Intracranial cortical and subcortical spectral biomarkers of sleep stages.
2. Intracranial cortical and subcortical spectral biomarkers of sleep dysfunction and awakening.

List any exploratory analyses that were not pre-specified and are included:

1. Time-resolved spectral power analyses,
2. Spectral coherence analyses
3. Correlation and cross-correlation analyses
4. Machine learning analyses

Is this an interim analysis (Y/N?): *No*

Informed written consent noted in the methods section (Y/N): *Yes*

For RCT: Parent trial daytime adaptive DBS– randomized double blind cross over trial
Sleep sub-study – NA observational.

Is a table presenting the patients baseline characteristics provided? *Yes (Table 1)*

If a statistical analysis plan is part of the study plan? *Yes*

Is the consort diagram provided? *Yes (Data flow and analysis included in Figure 1E)*
