# Supplementary material for: Multi-night cortico-basal recordings reveal mechanisms of NREM slow-wave suppression and spontaneous awakenings in Parkinson’s disease
Source: Nat Commun. 2024 Feb 27;15:1793. doi: 10.1038/s41467-024-46002-7 (PMC10899224; doi:10.1038/s41467-024-46002-7)
Supplement: Supplementary file 3 — Reporting Summary [file 41467_2024_46002_MOESM3_ESM.pdf]

nature portfolio

Corresponding author(s): Md Fahim Anjum

Last updated by author(s): Jan 30, 2024

Reporting Summary

Nature Portfolio wishes to improve the reproducibility of the work that we publish. This form provides structure for consistency and transparency in reporting. For further information on Nature Portfolio policies, see our Editorial Policies and the Editorial Policy Checklist.

Statistics

For all statistical analyses, confirm that the following items are present in the figure legend, table legend, main text, or Methods section.

n/a	Confirmed	
☐	☒	The exact sample size (*n*) for each experimental group/condition, given as a discrete number and unit of measurement
☐	☒	A statement on whether measurements were taken from distinct samples or whether the same sample was measured repeatedly
☐	☒	The statistical test(s) used AND whether they are one- or two-sided<br>*Only common tests should be described solely by name; describe more complex techniques in the Methods section.*
☐	☒	A description of all covariates tested
☐	☒	A description of any assumptions or corrections, such as tests of normality and adjustment for multiple comparisons
☐	☒	A full description of the statistical parameters including central tendency (e.g. means) or other basic estimates (e.g. regression coefficient) AND variation (e.g. standard deviation) or associated estimates of uncertainty (e.g. confidence intervals)
☐	☒	For null hypothesis testing, the test statistic (e.g. *F*, *t*, *r*) with confidence intervals, effect sizes, degrees of freedom and *P* value noted<br>*Give P values as exact values whenever suitable.*
☒	☐	For Bayesian analysis, information on the choice of priors and Markov chain Monte Carlo settings
☒	☐	For hierarchical and complex designs, identification of the appropriate level for tests and full reporting of outcomes
☒	☐	Estimates of effect sizes (e.g. Cohen's *d*, Pearson's *r*), indicating how they were calculated

Our web collection on statistics for biologists contains articles on many of the points above.

Software and code

Policy information about availability of computer code

Data collection

We collected data using two protocols: long-term multi-night data collection ON stimulation plus separate two night comparison recordings, one night ON DBS and one night OFF DBS. During the long-term overnight data collection, each participant (n=5) was equipped with a portable PSG (Dreem2) headset and overnight intracranial data as well as polysomnography data were recorded for ~10 nights that were predominantly consecutive. Extracranial polysomnography (PSG) was recorded through the Dreem2 headband which included an automated sleep staging algorithm with extracranial electroencephalography (EEG) data (Dreem2 headband, Dreem Co., Paris, France). The Dreem2 headband provided sleep stage classification hypnograms according to AASM scoring methods.

In the ON/OFF protocol, overnight data from the PD participants (n=4) were collected for two consecutive days. On the first day DBS was ON and the next day DBS was OFF. ON/OFF recordings were not completed in the cervical dystonia participant at the participant's request. During both data collection protocols, the PD participants were on their regular clinical dopaminergic replacement medications. All data recordings were performed remotely in participants' homes.

For each participant, the Summit RC+S device was implanted bilaterally and connected to bilateral sensing and stimulation-capable quadripolar leads in the basal ganglia targets (STN in 2 PD participants or GPi in 2 PD participants and 1 cervical dystonia participant) plus quadripolar sensorimotor chronic electrocorticography (ECoG), sensing only strips, with 4 electrode contacts spanning the central gyrus.

April 2023

Data analysis	All analyses were performed using Matlab 2021b (Mathworks). Codes generated and/or analyzed in this study will be shared upon reasonable request for reviewing purposes only. These codebases are part of the analyses of an ongoing medical research study. Publicly accessible code will be available upon the completion of the ongoing study at: https://github.com/MDFahimAnjum.

For manuscripts utilizing custom algorithms or software that are central to the research but not yet described in published literature, software must be made available to editors and reviewers. We strongly encourage code deposition in a community repository (e.g. GitHub). See the Nature Portfolio guidelines for submitting code & software for further information.

Data

Policy information about availability of data

All manuscripts must include a data availability statement. This statement should provide the following information, where applicable:

- Accession codes, unique identifiers, or web links for publicly available datasets
- A description of any restrictions on data availability
- For clinical datasets or third party data, please ensure that the statement adheres to our policy

All data supporting the findings of this study are available within the article and its supplementary files. Any additional requests for information can be directed to, and will be fulfilled by, the corresponding authors. The raw (identifiable) data from participants are privacy-protected. The processed de-identified data can only be shared on request as these datasets are part of an ongoing medical research study with active data collection and will subsequently be shared according to the National Institute of Health (NIH) data sharing requirements. Source data are provided as a Source Data file.

Research involving human participants, their data, or biological material

Policy information about studies with human participants or human data. See also policy information about sex, gender (identity/presentation), and sexual orientation and race, ethnicity and racism.

Reporting on sex and gender	We have 5 male participants. There were no bias during the recruitment process. Sex was determined by self reporting.
Reporting on race, ethnicity, or other socially relevant groupings	All 5 participants were Caucasian. All had bachelor of science (BS) and/ or masters (MA) degree except for one participant who had 3 years of college education.
Population characteristics	Our dataset of 5 participants had age of 55.4± 5 (mean ± SEM) years. We recruited 4 participants with idiopathic PD for this study. We also recruited one participant with cervical dystonia as a comparison participant. Participants were recruited from a parent study focused on investigating closed-loop DBS for daytime motor symptoms. For each participant, the Summit RC +S device was implanted bilaterally and connected to bilateral sensing and stimulation-capable quadripolar leads in the basal ganglia targets (STN in 2 PD participants or GPi in 2 PD participants and 1 cervical dystonia participant) plus quadripolar sensorimotor chronic electrocorticography (ECoG), sensing only strips, with 4 electrode contacts spanning the central gyrus. PD participants had UPDRS score of 48.75±6.1 (OFF) and 14.75±3.9 (ON).
Recruitment	In this study, we recruited 4 participants with idiopathic PD. A movement disorders physician diagnosed each individual with PD according to the Movement Disorder Society PD diagnostic criteria. The motor component of the United Parkinson's Disease Rating Scale (UPDRS) scores were administered by trained raters. We also recruited one participant with cervical dystonia as a comparison participant. These participants were recruited from a parent study focused on investigating closed-loop DBS for daytime motor symptoms. All participants had chronic bilateral cortical electrocorticography (ECoG) electrodes. Two PD participants were implanted with bilateral electrodes in the Subthalamic Nucleus and two PD participants along with one dystonia participant were implanted with bilateral electrodes in the Globus Pallidus nuclei. Implanted electrodes were connected to an investigational sensing-enabled Summit RC+S DBS implantable pulse generator provided by Medtronic. The cohort of this study included by definition patients undergoing DBS implantation which introduces a potential bias regarding the severity of the disease (PD/cervical dystonia) studied by including only those with relatively more advanced disease conditions.
Ethics oversight	This study was reviewed by our Institutional Review Board (University of California San Francisco Institutional Review Board) and registered on clinicaltrials.gov (NCT03582891; IDE G180097). The study was also reviewed by the Human Resources Protection Office (HRPO) at Defense Advanced Research Projects Agency (DARPA). This study was conducted in accordance with the Declaration of Helsinki. All participants provided informed written consent for participation in the study and publishing of their de-identified data. No direct compensation was provided to the participants for participating in the study.

Note that full information on the approval of the study protocol must also be provided in the manuscript.

Field-specific reporting

Please select the one below that is the best fit for your research. If you are not sure, read the appropriate sections before making your selection.

☒ Life sciences ☐ Behavioural & social sciences ☐ Ecological, evolutionary & environmental sciences

For a reference copy of the document with all sections, see nature.com/documents/nr-reporting-summary-flat.pdf

Life sciences study design

All studies must disclose on these points even when the disclosure is negative.

Sample size	Our sample size was 4 PD patients and 1 cervical dystonia patient. The sample size was determined by the availability of participants willing to join the study. Primary analyses were powered to be completed at the within subject level. This study did not contain a sample size calculation as there was no available human pilot data on which to based effect sizes. However, it is noted that given the ~10 day overnight data recording per participant with a total of 407 hours of sleep data using 5-30 second data epochs investigated, this study was highly statistically powered at the individual subject level. Linear mixed effect models were then used to combine data across subjects looking for group level effects.
Data exclusions	Exclusion Criteria<br><br>Parkinson's Disease:<br>1. Coagulopathy, anticoagulant medications, uncontrolled hypertension, history of seizures, heart disease, or other medical conditions considered to place the patient at elevated risk for surgical complications<br>2. Evidence of a psychogenic movement disorder: Motor symptoms that remit with suggestion or "while unobserved", symptoms that are inconsistent over time or incongruent with clinical condition, plus other manifestation such as "false" signs, multiple somatizations, or obvious psychiatric disturbance.<br>3. Pregnancy: all women of child bearing potential will have a negative urine pregnancy test prior to undergoing their surgical procedure.<br>4. Significant untreated depression (BDI-II score >20) History of suicidal attempt or active suicidal ideation (Yes to #2-5 on C-SSRS)<br>5. Any personality or mood symptoms that study personnel believe will interfere with study requirements.<br>6. Subjects who require ECT, rTMS or diathermy<br>7. Implanted stimulation systems such as; cochlear implant, pacemaker, defibrillator, neurostimulator or metallic implant<br>8. Previous cranial surgery<br>9. Drug or alcohol abuse<br>10. Meets criteria for Parkinson's disease with mild cognitive impairment (PD-MCI). These criteria are: performance of more than two standard deviations below appropriate norms, for tests from two or more of these five cognitive domains: attention, executive function, language, memory, and visuospatial tests.<br><br>Dystonia:<br>1. Coagulopathy, anticoagulant medications, uncontrolled hypertension, history of seizures, heart disease, or other medical conditions considered to place the patient at elevated risk for surgical complications<br>2. Evidence of a psychogenic movement disorder: Motor symptoms that remit with suggestion or "while unobserved", symptoms that are inconsistent over time or incongruent with clinical condition, plus other manifestation such as "false" signs, multiple somatizations, or obvious psychiatric disturbance.<br>3. Pregnancy: all women of child bearing potential will have a negative urine pregnancy test prior to undergoing their surgical procedure.<br>4. Significant untreated depression (BDI-II score >20) History of suicidal attempt or active suicidal ideation (Yes to #2-5 on C-SSRS)<br>5. Any personality or mood symptoms that study personnel believe will interfere with study requirements.<br>6. Subjects who require ECT, rTMS or diathermy<br>7. Implanted stimulation systems such as; cochlear implant, pacemaker, defibrillator, neurostimulator or metallic implant<br>8. Previous cranial surgery<br>9. Drug or alcohol abuse<br><br>We initially collected a total of 58 nights of data. Upon manual inspection, we rejected a recording from one night which had 95% missing intracranial data.
Replication	In addition to 5-fold cross-validation, we also implemented a 2-fold (50% of the data were used for training and the remaining data were tested) cross-validation scheme to investigate the robustness of our performance.
Randomization	This was an observational study and therefore no randomization was completed.
Blinding	The study was observational and unblinded. Note for sleep neurophysiology we had objective demonstration that the patients were asleep from Polysomnogram recordings

Reporting for specific materials, systems and methods

We require information from authors about some types of materials, experimental systems and methods used in many studies. Here, indicate whether each material, system or method listed is relevant to your study. If you are not sure if a list item applies to your research, read the appropriate section before selecting a response.

Materials & experimental systems

n/a	Involved in the study
☒	☐ Antibodies
☒	☐ Eukaryotic cell lines
☒	☐ Palaeontology and archaeology
☒	☐ Animals and other organisms
☐	☒ Clinical data
☒	☐ Dual use research of concern
☒	☐ Plants

Methods

n/a	Involved in the study
☒	☐ ChIP-seq
☒	☐ Flow cytometry
☒	☐ MRI-based neuroimaging

Clinical data

Policy information about clinical studies

All manuscripts should comply with the ICMJE guidelines for publication of clinical research and a completed CONSORT checklist must be included with all submissions.

Clinical trial registration: https://clinicaltrials.gov/study/NCT03582891

Study protocol: https://clinicaltrials.gov/study/NCT03582891

Data collection: We recruited 4 participants with idiopathic PD for this study. We also recruited one participant with cervical dystonia as a comparison participant. Participants were recruited from a parent study focused on investigating closed-loop DBS for daytime motor symptoms. The First participant enrolment for this study was in February 2021 and the last enrolment was in June 2021. A movement disorders physician diagnosed each individual with PD according to the Movement Disorder Society PD diagnostic criteria. The motor component of the United Parkinson's Disease Rating Scale (UPDRS) scores were administered by trained raters.

All participants had chronic bilateral cortical ECoG electrodes and two PD participants were implanted with bilateral electrodes in the Subthalamic Nucleus and two PD participants along with one dystonia participant were implanted with bilateral electrodes in the Globus Pallidus nuclei. DBS electrode implantation targets were determined by the clinical team. Implanted electrodes were connected to an investigational sensing-enabled Summit RC+S DBS implantable pulse generator provided by Medtronic. A movement disorder specialist programmed the participants with conventional DBS settings, optimizing stimulation to address daytime motor symptoms.

We collected data using two protocols: long-term multi-night data collection ON stimulation plus separate two night comparison recordings, one night ON DBS and one night OFF DBS. During the long-term overnight data collection, each participant (n=5) was equipped with a portable PSG (Dreem2) headset and overnight intracranial data as well as polysomnography data were recorded for ~10 nights that were predominantly consecutive. In the ON/OFF protocol, overnight data from the PD participants (n=4) were collected for two consecutive days. On the first day DBS was ON and the next day DBS was OFF. ON/OFF recordings were not completed in the cervical dystonia participant at the participant's request. During both data collection protocols, the PD participants were on their regular clinical dopaminergic replacement medications. All data recordings were performed remotely in participants' homes.

Outcomes: Outcomes of Sleep Study:
Primary outcome: Subcortical beta - cortical delta interaction during NREM sleep.
Secondary outcome: Change of subcortical beta power prior to awakenings in PD

Primary outcome was measured by segmenting multi-night at-home intracranial recordings of the participants during NREM sleep in 5s data epochs, calculating subcortical beta power as well as cortical delta power for these data epochs, and constructing linear mixed effect models (LME) models for cortical delta power with subcortical beta power and disease state as fixed effects and hemisphere (left/right), participants, night# as random effects. The LME models measured the subcortical beta - cortical delta interaction during NREM sleep.

Secondary outcome was measured by investigating data from all NREM to wakefulness sleep transitions in our multi-night at-home intracranial recordings of the PD participants. Events with NREM sleep less than 85s and wake periods of less than 25s were ignored. Beta power for each 5s data epoch was averaged. All epochs in N2/N3 NREM data after 40s from NREM onset and 40s before awakening were averaged to calculate deep NREM power. Finally, LME models were constructed for beta power with deep NREM vs pre/post awakening state and disease state as fixed and participants as random effects. The LME models assessed the change of subcortical beta power prior to awakenings in PD.

Plants

Seed stocks	*Report on the source of all seed stocks or other plant material used. If applicable, state the seed stock centre and catalogue number. If plant specimens were collected from the field, describe the collection location, date and sampling procedures.*
Novel plant genotypes	*Describe the methods by which all novel plant genotypes were produced. This includes those generated by transgenic approaches, gene editing, chemical/radiation-based mutagenesis and hybridization. For transgenic lines, describe the transformation method, the number of independent lines analyzed and the generation upon which experiments were performed. For gene-edited lines, describe the editor used, the endogenous sequence targeted for editing, the targeting guide RNA sequence (if applicable) and how the editor was applied.*
Authentication	*Describe any authentication procedures for each seed stock used or novel genotype generated. Describe any experiments used to assess the effect of a mutation and, where applicable, how potential secondary effects (e.g. second site T-DNA insertions, mosiacism, off-target gene editing) were examined.*
